# Work-Related Musculoskeletal Disorders and Occupational Burnout Among Pet Grooming Professionals: Prevalence, Ergonomic Risk Factors, and Their Interrelationships

**DOI:** 10.3390/healthcare14142213

**Published:** 2026-07-21

**Authors:** Su-Hui Cheng, Mei-Ru Chen, Chung-Jung Tsai

**Affiliations:** Department of Occupational Safety and Health, Chung Hwa University of Medical Technology, Tainan City 717302, Taiwan; chenmj@mail.hwai.edu.tw

**Keywords:** pet groomers, musculoskeletal disorders, ergonomic risk factors, occupational burnout, repetitive work, occupational health

## Abstract

**Highlights:**

**What are the main findings?**

- Work-related musculoskeletal disorders affected 95% of pet grooming professionals, with the shoulders, neck, upper back, and lower back being the most vulnerable body regions.
- Moderate-to-severe occupational burnout was common and was significantly associated with musculoskeletal disorder severity.

**What are the implications of the main findings?**

- Ergonomic redesign of grooming workstations and wider adoption of adjustable equipment may substantially reduce the burden of musculoskeletal disorders.
- Occupational health programs targeting both physical workload and psychological well-being should be integrated into the pet grooming industry.

**Abstract:**

**Background/Objectives**: The rapid growth of the companion animal industry has increased demand for professional pet grooming services; however, occupational health risks among pet groomers remain underexplored. This cross-sectional study investigated the prevalence of musculoskeletal disorders (MSDs), occupational burnout, and associated occupational and ergonomic factors and their interrelationships among pet grooming professionals. **Methods**: A total of 180 pet grooming professionals were recruited through geographically stratified convenience sampling across northern, central, and southern Taiwan. Data were collected using structured questionnaires assessing demographics, workload, ergonomic equipment, and working postures. MSD symptoms were evaluated using the Nordic Musculoskeletal Questionnaire, and occupational burnout was assessed using the Taiwan Ministry of Labor Burnout Assessment Scale. Descriptive statistics, one-way analysis of variance (ANOVA), Pearson’s correlation analysis, and multiple linear regression analysis were performed to identify factors associated with MSD severity and occupational burnout. **Results**: MSDs were highly prevalent, with 95% of participants reporting symptoms in at least one body region. The most affected regions were the right shoulder (71.7%), neck (70.6%), upper back (65.6%), and lower back (65.6%). Significant associated ergonomic factors included excessive bathing tub depth (≥46 cm), non-adjustable grooming tables, prolonged trunk flexion, and horse-stance postures. Higher workload intensity, including frequent bathing tasks and handling aggressive or large animals, was significantly associated with greater MSD severity (*p* < 0.05). Moderate-to-severe personal- and work-related burnout were identified in 43.3% and 37.2% of participants, respectively. MSD severity was positively correlated with personal-related burnout (r = 0.491, *p* < 0.001) and work-related burnout (r = 0.428, *p* < 0.001). Multiple linear regression analysis results indicated the MSD score was a significant predictor of both personal- and work-related burnout (*p* < 0.001). **Conclusions**: Pet grooming professionals experience a substantial burden of MSDs and occupational burnout associated with ergonomic strain and physically demanding work. Non-neutral postures and inadequately designed equipment were significantly associated with greater MSD severity. The close association between MSDs and burnout underscores the interrelationship between physical and psychological occupational health. Ergonomic workplace modifications and targeted occupational health interventions are necessary to improve worker well-being and industry sustainability.

## 1. Introduction

The global trend of “pet humanization” has fundamentally transformed the companion animal industry. Driven by demographic shifts, such as aging populations and declining birth rates, pets are increasingly regarded as integral family members rather than mere property [1]. This paradigm shift has catalyzed a booming “pet economy,” expanding beyond basic necessities to include specialized healthcare, luxury products, and aesthetic services [2]. Consequently, the pet grooming sector has experienced rapid professionalization and growth. In Taiwan, the number of registered grooming businesses has surged, reflecting a high demand for services ranging from basic hygiene—such as ear cleaning and nail trimming—to complex styling and bathing procedures [3].

Despite the economic vitality of this sector, the occupational health hazards associated with pet grooming remain under-researched. Pet grooming is a physically demanding profession characterized by a unique combination of ergonomic risks found in related fields: the repetitive upper-limb movements of hairdressers and the heavy lifting and physical restraint required of veterinary professionals [4,5]. Groomers frequently perform tasks requiring awkward postures, such as prolonged trunk flexion over bathtubs, static standing, and forceful exertion to manage uncooperative animals [6].

Recent studies indicate that veterinary professionals and technicians exhibit the prevalence rates of musculoskeletal disorders (MSDs) exceeding 60%, particularly in the lower back and neck, attributed to patient handling and surgical postures [7,8]. Similarly, hairdressers report MSD prevalence rates ranging from 66% to 75%, primarily driven by prolonged standing and repetitive arm movements [9,10]. However, pet groomers face a compounded risk: they must navigate the precise dexterity required for scissoring while simultaneously managing the dynamic and unpredictable physical loads of live animals [4,6]. Furthermore, the profession necessitates highly repetitive manual tasks, such as deshedding, shearing, bathing, and blow-drying, which predispose practitioners to upper-extremity repetitive strain injuries (RSIs) and tendinopathy. These risks are compounded by environmental and ergonomic factors; working in confined spaces, maintaining non-neutral postures (e.g., sustained bending, twisting, or kneeling), and prolonged standing on unyielding surfaces cumulatively exacerbate pressure on the axial skeleton and lower extremities, culminating in both physical and cognitive fatigue. Additionally, inadequate recovery periods, coupled with the use of suboptimal or poorly maintained equipment, further contribute to occupational overexertion [11,12].

Musculoskeletal disorders (MSDs) now constitute a significant socioeconomic burden. Due to an aging population, MSDs have become a growing health and economic burden in the United States. In 2019, MSDs affected approximately 127.4 million people (more than one-third of the U.S. population); they were the largest driver of healthcare spending in 2016, with estimated direct costs of $380.9 billion annually. MSDs constitute a substantial and growing burden in terms of prevalence, disability, and medical costs [13]. Data from Taiwan’s Labor Insurance Bureau shows that in 2020, claims for MSDs among workers reached NT$16.9 billion (approximately US$0.57 billion), accounting for 52% of all occupational disease claims [14]. Occupational burnout is increasingly recognized as a major occupational health concern extending beyond psychological well-being. It is characterized by persistent physical, emotional, and mental exhaustion resulting from chronic work-related stress and has been associated with reduced work performance, impaired concentration and decision-making, increased absenteeism, and diminished quality of life [15,16,17,18,19]. Previous studies have further suggested that burnout may coexist with chronic musculoskeletal pain through shared occupational exposures, including prolonged working hours, repetitive tasks, high physical workload, insufficient recovery, and adverse psychosocial working conditions [15,16,17,18,19]. Furthermore, accumulating evidence indicates that psychosocial factors, such as high job demands, low job control, inadequate organizational support, and emotional workload, may interact with biomechanical exposures to increase the risk of both work-related musculoskeletal disorders (WMSDs) and occupational burnout [19,20,21]. Nevertheless, despite the rapid expansion of the pet grooming industry, research investigating occupational burnout and its relationship with musculoskeletal disorders in this profession remains extremely limited. Most available studies have focused primarily on biological hazards, injuries, or ergonomic risks [11,12,22], while the psychological dimensions of occupational health have received considerably less attention. To our knowledge, this is one of the first studies to comprehensively investigate work-related musculoskeletal disorders, occupational burnout, ergonomic workplace characteristics, workload, working postures, and their interrelationships among pet grooming professionals. Therefore, the objectives of this study were to determine the prevalence of MSDs and occupational burnout, identify occupational and ergonomic factors associated with these outcomes, and explore the relationships between musculoskeletal disorders and occupational burnout. The findings are expected to provide evidence for the development of targeted ergonomic interventions and occupational health strategies for this rapidly growing profession.

## 2. Methods

### 2.1. Participants and Study Design

This study focused on pet grooming professionals, including pet groomers, grooming administrators, grooming assistants, and veterinary assistants involved in grooming tasks. Participants were required to be at least 20 years old and possess a minimum of one year of full-time work experience. A total of 180 volunteers were recruited to participate in the study by geographically stratified convenience sampling, including 58 subjects from north, 58 subjects from middle and 64 subjects from south of Taiwan. An a priori sample size calculation was performed using G*Power version 3.1.9.7 (Heinrich-Heine-University Düsseldorf, Germany). Assuming a medium effect size (f = 0.25), a significance level of α = 0.05, statistical power of 0.95, and one-way analysis of variance as the primary statistical analysis, the minimum required sample size was estimated to be 172 participants. To compensate for potential non-response and incomplete questionnaires, the target sample size was increased to 180 participants, all of whom completed valid questionnaires and were included in the final analyses.

Prior to data collection, the research protocol and questionnaire were reviewed and approved by the National Cheng Kung University Governance Framework for Human Research Ethics (Review Number: NCKU HREC-E-108-438-2) to ensure the protection of participant privacy and rights. All participants had read the approved study instructions carefully before the study and voluntarily completed anonymous questionnaires.

### 2.2. Instruments

The study employed a comprehensive survey comprising demographic and occupational characteristics, work equipment specifications, workload metrics, and working postures, alongside the musculoskeletal and work fatigue assessment. Demographic and occupational characteristics were collected, including gender, age, height, weight, body mass index (BMI), years of service, daily working hours, and the specific types of animal species handled. Work equipment specifications were evaluated, focusing on the physical dimensions of the bathing tub (specifically its depth and height), the adjustability of grooming table heights, and the specific configurations of the blow dryers utilized. Workload metrics were quantified by recording the maximum and average daily bathing volumes, the specific daily volume of small dogs, and the weekly average volumes of medium-to-large dogs, aggressive animals, and both manual and electric trimming tasks. Typical working postures were systematically assessed during three core professional tasks: seated blow-drying (categorized as upright/neutral, neck flexion, or back flexion), manual trimming (categorized as seated, lunge, standing upright with straight knees, or horse stance), and electric clipping (categorized as seated, lunge, standing upright, or horse stance).

Demographic and Occupational Characteristics: Gender, age, height, weight, BMI, years of service, daily working hours, and types of animals handled.

Work Equipment Specifications: Dimensions of the bathing tub (depth and height), grooming table height, and type of blow dryer used.

Workload Metrics: Maximum and average daily bathing volume, specific volume of small dogs, and weekly average volumes for medium/large dogs, aggressive animals, and trimming tasks (manual and electric).

Working Postures: Assessed during three key tasks:

Blow-drying (Seated): Upright (neutral), neck flexion/head down, or back flexion.

Manual Trimming: Seated, lunge, standing upright (knees straight), or horse stance (wide squat).

Electric Clipping: Seated, lunge, standing upright (knees straight), or horse stance.

**Figure 1:**
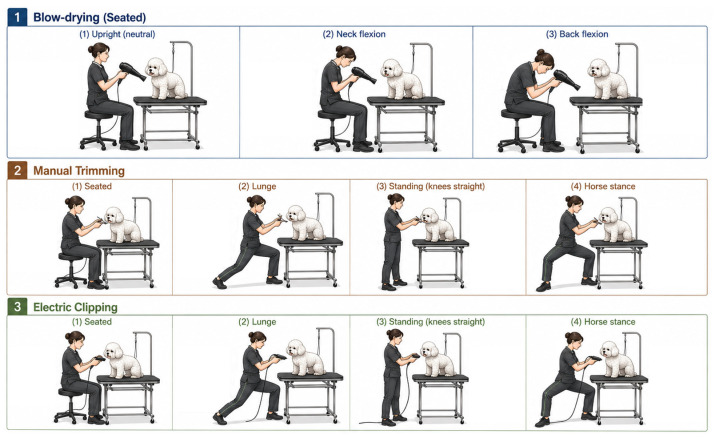
Typical Working Postures of Pet Grooming Professionals.

The typical working postures are presented in Figure 1.

Musculoskeletal Disorders Assessment: Work-related musculoskeletal symptoms were assessed using the standardized Nordic Musculoskeletal Questionnaire (NMQ), an internationally validated instrument for epidemiological studies of musculoskeletal disorders. The questionnaire evaluates the presence of musculoskeletal symptoms in 15 anatomical regions, including the neck, shoulders, upper back, lower back, elbows/forearms, wrists/hands, hips/thighs, knees, and ankles/feet, during the preceding 12 months. Participants reported whether they had experienced pain or discomfort in each body region, and the total number of symptomatic body regions was calculated as the musculoskeletal disorder (MSD) score, which was subsequently used as a continuous variable in the statistical analyses. The pain score for each body region in the NMQ was quantified using a 6-point ordinal scale (0–5), with scores ranging from 0 (no pain) to 5 (very severe pain). The NMQ has demonstrated excellent reliability (77–100%) and validity (80–100%) across occupational populations and is widely used in occupational epidemiological research [23,24].

Occupational Burnout Assessment: The “Burnout Assessment Scale” developed by the Institute of Occupational Safety and Health, Taiwan Ministry of Labor, includes two sections: “Personal-Related Burnout” and “Work-Related Burnout”. Personal-related burnout includes 6 items that measure generic physical, emotional, and mental fatigue perceived by individuals regardless of their employment status. Work-related burnout includes 7 items that assess symptoms of fatigue and exhaustion explicitly attributed to the respondent’s current employment conditions. The “Burnout Assessment Scale” was adapted from the Copenhagen Burnout Inventory (CBI) and underwent comprehensive construct validation and cross-sectional calibration using data from large-scale, nationwide employee surveys in Taiwan. Cronbach’s alpha (α) coefficients were reported at 0.92 for the personal-related burnout scale and 0.89 for the work-related burnout scale, indicating robust scale reliability. Personal-related burnout scores are categorized as follows: below 50 points indicates mild, 50–70 points indicates moderate, and above 70 points indicates severe burnout. Work-related burnout scores are categorized as follows: below 45 points indicates mild, 45–60 points indicates moderate, and above 60 points indicates severe burnout [25].

All collected questionnaires were checked for completeness, coded, and entered into a computerized database for statistical analysis using Microsoft Excel and IBM SPSS Statistics (Version 27.0; IBM Corp., Armonk, NY, USA). A two-tailed *p*-value of <0.05 was considered indicative of statistical significance. Continuous variables are presented as means ± standard deviations, whereas categorical variables are expressed as frequencies and percentages. One-way analysis of variance (ANOVA) was used to compare MSD scores among different occupational and ergonomic exposure groups, followed by post hoc comparisons when appropriate. Pearson’s correlation analysis was conducted to examine associations between continuous variables and burnout scores. To identify independent predictors of personal-related burnout and work-related burnout, multiple linear regression analyses were performed after adjustment for potential confounding variables, including age, BMI, years of service, daily working hours, daily standing hours, daily sitting hours, and MSD score.

## 3. Results

### 3.1. Demographics and Occupational Characteristics

Table 1 presents the demographic profile of the 180 participants. The workforce was predominantly female (83.3%). The mean age was 33.8 ± 8.8 years, with the majority (74.4%) aged between 20 and 39 years, indicating a younger workforce. The average height was 162.2 ± 7.0 cm, and the average weight was 59.4 ± 11.7 kg; 28.9% of participants had a BMI classified as overweight or obese (>24).

Participants had an average of 8.2 ± 7.3 years of service. Daily working hours averaged 8.9 ± 1.5 h, with 82.8% working 8–10 h daily. Regarding the animal species handled (multiple responses permitted), 179 participants (99.4%) reported grooming dogs, 106 (58.9%) groomed cats, and 110 (61.1%) routinely groomed both dogs and cats. Because participants could select more than one category, these percentages are not mutually exclusive and therefore do not total 100%.

### 3.2. Equipment and Workload Analysis

Table 2 details the equipment specifications. The most common bathing tub depth was 36–45 cm (38.9%), followed by 26–35 cm (35.6%). Regarding tub height, 53.3% of units were positioned between the operator’s waist and chest. The bathing tubs in Taiwan were almost fixed units. This lack of tub adjustability forces the operator to adapt their posture entirely to the fixed basin dimensions, regardless of their own anthropometry or the animal’s size. Nearly half (48.9%) of the grooming tables were fixed at a height of 75–79 cm, while only 28.3% were height-adjustable. Upright stand dryers were the most prevalent equipment (63.9%).

Table 3 illustrates the workload metrics. While the average daily bathing volume was typically ≤15 pets (97.2%), peak daily volumes often reached 10–20 pets (56.7%) or 21–30 pets (28.3%). Given that washing and drying a single pet takes approximately 70 min [26], these volumes suggest significant time pressure. Furthermore, the handling of medium/large dogs and aggressive animals contributes additional physical load and mental stress.

### 3.3. Musculoskeletal Disorders and Risk Factors

An alarmingly high 95% of participants reported musculoskeletal disorders in at least one body region over the past year. Table 4 ranks the prevalence of symptoms by body part. The most affected areas were the right shoulder (71.1%), neck (70.6%), upper back (65.6%), and lower back (65.6%). The data indicates a concentration of disorders in the upper torso and the dominant (right) hand.

Table 5 analyzes the relationship between equipment specifications and high-frequency pain areas (>70% prevalence).

Bathing Tub Depth: Tubs deeper than 46 cm were associated with severe pain (>70% prevalence) in the lower back, neck, right wrist, and right shoulder. Tubs with a depth of 36–45 cm (approximate elbow-to-fingertip length) appeared to be the most ergonomic, with only the neck showing >70% pain prevalence.

Bathing Tub Height: Heights between waist and chest levels were associated with high prevalence of pain in the right shoulder (78.1%), neck (74.0%), and upper back (71.9%). The study suggests the optimal height is at or below the waist.

Grooming Table Height: Fixed tables generally correlated with high neck and shoulder pain. Notably, adjustable tables resulted in only one region (lower back) exceeding 70% prevalence, suggesting they are the superior ergonomic choice.

Table 6 analyzes pain prevalence relative to working postures.

Blow-drying: The “Back flexion” posture was most damaging, with neck pain reaching 79.6%.

Manual Trimming: The “Horse Stance” (wide squat) associated with the highest right shoulder pain (83.8%), while standing upright with locked knees associated with high pain across the upper back and shoulders. The “Lunge” position appeared relatively safer but still resulted in 67.2% neck pain.

Electric Clipping: All postures showed high risks, but the “Horse Stance” was particularly detrimental to the right shoulder (84.6%). Standing upright was comparatively the least painful posture for this task.

Table 7 demonstrates significant correlations between workload intensity and MSD severity. Higher daily bathing volumes (>21 pets) and handling large numbers of small dogs were strongly correlated with increased pain scores (*p* < 0.001 and *p* = 0.001, respectively). Handling aggressive animals also showed a significant correlation (*p* = 0.041).

### 3.4. Occupational Burnout, Risk Factors and the Association with MSDs

Table 8 presents the descriptive analysis of burnout levels among the pet grooming professionals. Regarding Personal-related Burnout, 14.4% of respondents reported “Severe” levels, while 28.9% experienced “Moderate” fatigue. Similarly, in the Work-related Burnout dimension, 12.2% of the cohort reached the “Severe” threshold, and 25.0% were classified as “Moderate”. Collectively, these results indicate that over one-third of pet grooming professionals exhibit clinically significant levels of burnout, categorized as moderate to severe.

To identify the key drivers of occupational burnout, a Pearson correlation analysis was conducted to examine the relationships between burnout levels and various demographic and occupational variables. The analysis focused on age, years of service, daily standing duration, daily working hours, daily sitting duration, and musculoskeletal disorder. Table 9 highlights several critical correlations. Personal-related burnout demonstrated a statistically significant positive correlation with daily working hours (*p* = 0.035), daily sitting duration (*p* = 0.003), and musculoskeletal disorder (*p* < 0.001). Work-related burnout showed a significant positive correlation with daily sitting duration (*p* = 0.013) and musculoskeletal disorder (*p* < 0.001). Musculoskeletal disorder presented as the strongest correlation for both personal-related (r = 0.491) and work-related (r = 0.428) burnout. Conversely, variables such as age, years of service, and daily standing duration did not show a statistically significant relationship with burnout levels.

Multiple regression statistical analysis was used to explore the factors influencing occupational burnout for the pet grooming professionals in this study. The demographic and occupational variables, including age, years of service, daily standing duration, daily working hours, daily sitting duration, and musculoskeletal disorder were used as independent variables, and the dependent variable was the personal-related burnout score and the work-related burnout score, respectively. The results presented in Table 10 indicated that both daily sitting hours and the MSD score were significantly associated with the personal-related burnout score and the work-related burnout score (both *p* < 0.05). Notably, the MSD score demonstrated a particularly strong association with both dimensions of occupational burnout (both *p* < 0.001). These findings suggest that the high prevalence and severity of musculoskeletal disorder among pet grooming professionals are closely linked to increased levels of occupational burnout. Specifically, greater musculoskeletal symptom burden appears to exert a substantial impact on both personal- and work-related burnout, underscoring the important role of musculoskeletal health in the psychological well-being of this occupational group.

## 4. Discussion

The present study provides one of the first comprehensive investigations of work-related musculoskeletal disorders (MSDs), occupational burnout, ergonomic workplace characteristics, workload, and working postures among pet grooming professionals. Although the companion animal industry has expanded rapidly worldwide, occupational health research in this profession remains limited, with previous studies focusing primarily on biological hazards, injuries, or isolated ergonomic issues. By simultaneously examining physical workload, workplace ergonomics, musculoskeletal symptoms, and occupational burnout within a single analytical framework, this study provides novel scientific evidence regarding the occupational health challenges faced by pet grooming professionals and offers a foundation for future ergonomic intervention and longitudinal research. This study reveals a high prevalence of work-related musculoskeletal disorders (MSDs) among pet groomers, with 95% of participants reporting symptoms. This figure notably exceeds recent prevalence rates reported in comparable professions. For instance, recent epidemiological studies have documented MSD prevalence rates of approximately 60–73% among veterinarians and veterinary technicians in the United States [7] and 66–76% among hairdressers in South Asia [9,10]. The higher prevalence observed in this study suggests that pet groomers may be exposed to a synergistic effect of risk factors: the cumulative trauma of repetitive fine-motor tasks (akin to hairdressing) combined with the high-force requirements of lifting and restraining animals (akin to veterinary work).

A critical finding of this study is the significant association between equipment dimensions—specifically bathing tub depth and grooming table height—and musculoskeletal injury. Our results corroborate biomechanical principles suggesting that working surfaces unsuited to anthropometric dimensions force workers into non-neutral postures. Standard ergonomic guidelines for animal care recommend that working heights be adjustable to waist level to minimize trunk flexion [27]. However, our data indicate that deep bathing tubs force groomers into excessive forward flexion and shoulder abduction to reach the animal, significantly increasing the moment arm on the lumbar spine. This is consistent with findings by comparable studies in veterinary surgery, where static, awkward postures were primary predictors of spinal pain [8]. However, biomechanical exposure is primarily governed by the effective working height, which is determined by the interaction between worker anthropometry (particularly elbow height), bathtub height and depth, animal size and posture, and the use of any auxiliary support devices. Consequently, identical bathtub dimensions may result in substantially different trunk flexion angles and shoulder loading depending on the size of the animal being groomed. For example, bathing a small dog in a deep bathtub generally requires greater forward trunk flexion than bathing a large dog, whereas the use of an elevated platform inside the bathtub may effectively improve working posture. Therefore, bathtub dimensions should be interpreted as surrogate ergonomic indicators rather than independent causal factors.

Furthermore, the dynamic nature of the workload distinguishes pet grooming from handling inanimate objects. The “live load” of an animal introduces unpredictability; sudden movements during bathing or nail trimming require groomers to exert rapid, reactive stabilizing forces, exacerbating the risk of acute strain on top of chronic fatigue [6]. The prevalence of wrist and shoulder injuries in our sample also aligns with the high frequency of repetitive movements required for scissoring and brushing, a risk factor shared with the hairdressing industry but intensified by the need to stabilize the animal [5,28]. This study also highlights the potential role of gender and workforce demographics. With the industry being predominantly female, equipment design standards may not adequately accommodate the average female groomer’s stature, leading to further ergonomic mismatch.

While occupational burnout is a near-ubiquitous phenomenon among pet grooming professionals, excessive occupational burnout establishes a critical juncture where the interplay between health, safety, and productivity can precipitate a detrimental “vicious cycle.” The most salient impacts of occupational burnout include attenuated work motivation, delayed reaction times, and diminished vigilance. Furthermore, it disrupts cognitive functions such as concentration, psychomotor coordination, memory, information processing, and executive judgment [29]. Escalating burnout levels further correlate with impaired mood, reduced job satisfaction, and a marked deterioration in alertness and coordination [30,31,32]. Beyond immediate performance, burnout is associated with chronic health sequelae—including gastrointestinal disorders, cardiovascular disease, psychological stress, and mental illness—and is a significant predictor of increased absenteeism [30]. Existing literature attributes occupational burnout to factors such as prolonged working hours, suboptimal environmental conditions, and high workloads [17]. Consistent with these findings, the present study identifies that over one-third of pet grooming professionals experience moderate-to-severe occupational burnout, driven by specific stressors including extended shifts, inadequate equipment, and intensive workloads. Notably, this research explores the nexus between occupational burnout and MSDs—a relationship seldom addressed in previous studies of this cohort. The results demonstrate a high degree of correlation between burnout and MSDs. To mitigate these occupational hazards, interventions should prioritize addressing the risks associated with prolonged work and sedentary. This study has several limitations that should be considered when interpreting the findings. First, due to the cross-sectional nature of this study, a direct causal relationship between MSDs and occupational burnout cannot be definitively established. An alternative explanation is that both outcomes simultaneously manifest from a shared underlying pattern of severe occupational exposure—namely prolonged working hours, repetitive tasks, and high cumulative workload. This common exposure pathway likely drives both somatic degradation and psychological exhaustion concurrently. Nevertheless, our multiple linear regression model offers deeper insight into this relationship. Even after adjusting for key exposure metrics such as daily working hours, standing duration, and sitting hours, the MSD score remained a robust, independent predictor of both personal- and work-related burnout (*p* < 0.001). This suggests that while shared work stressors act as a foundational cause, the persistent somatic burden of musculoskeletal pain may independently exacerbate psychological distress. Second, although participants were recruited through a nationwide survey of pet grooming professionals in Taiwan, caution should be exercised when generalizing the results to populations with different occupational environments or cultural backgrounds. Future studies employing longitudinal or prospective designs and involving participants from multiple countries are warranted to validate and extend the present findings. Such research is particularly needed in Asian regions, where evidence concerning the relationship between musculoskeletal disorders and occupational burnout among pet grooming professionals remains limited. In addition, biomechanical workload during pet grooming is influenced by numerous animal-related characteristics, including body weight, breed, coat condition, grooming duration, handling difficulty, and behavioral responses such as aggressiveness. Likewise, workplace factors—including lifting assistance from owners or coworkers, the availability of ramps or mechanical lifting devices, and organizational work practices—may substantially modify physical workload. These variables should be incorporated into future research to enable more precise exposure assessment.

## 5. Conclusions

Pet grooming professionals experience a substantial burden of work-related musculoskeletal disorders and occupational burnout, with the shoulders, neck, upper back, and lower back being the most affected regions. Ergonomic risk factors—including excessive bathing tub depth, non-adjustable grooming tables, prolonged trunk flexion, and non-neutral working postures—were significantly associated with increased musculoskeletal symptoms. In addition, high workload intensity and frequent handling of large or aggressive animals further exacerbated MSD severity.

A significant positive relationship was observed between musculoskeletal disorders and both personal-related and work-related burnout, indicating a close interaction between physical and psychological occupational strain. These findings highlight the urgent need for industry-specific occupational health interventions, particularly ergonomic workstation redesign, adjustable equipment, posture training, workload management, and preventive health surveillance.

As the companion animal industry continues to expand globally, improving occupational health conditions in the pet grooming profession is essential for protecting worker well-being, reducing injury risk, and sustaining long-term workforce productivity. Future research directions could be expanded to include longitudinal studies, objective ergonomic assessments, and intervention-based research.

## Figures and Tables

**Table 1:** Analysis of demographic and occupational characteristics of pet grooming professionals.

Variable	Item	Sample Size (*N*)	Percentage (%)
Gender	Male	30	16.7
	Female	150	83.3
Age	20–29 years	72	40
	30–39 years	62	34.4
	40–49 years	32	17.8
	50–59 years	14	7.8
	33.8 ± 8.8 years ^b^		
Height	150–160 cm	85	47.2
	161–170 cm	71	39.5
	17.1–180 cm	24	13.3
	162.2 ± 7.0 cm ^b^		
Weight	40–50 kg	47	26.1
	51–60 kg	69	38.3
	61–70 kg	41	22.6
	71–80 kg	14	7.8
	81–90 kg	5	2.8
	>90 kg	4	2.2
	59.4 ± 11.7 kg ^b^		
BMI	<18.5	16	8.9
	18.5–24	112	62.2
	>24	52	28.9
	22.6 ± 3.9 ^b^		
Years of service	1–4.9 years	69	38.3
	5–9.9 years	57	31.7
	10–14.9 years	23	12.8
	15–19.9 years	16	8.9
	≥20 years	15	8.3
	8.2 ± 7.3 years ^b^		
Daily working hours	<8 h	17	9.4
	8–10 h	149	82.8
	>10 h	14	7.8
	8.9 ± 1.5 h ^b^		
Animal species handled ^a^	dog	179	99.4
	cat	106	58.9
	dog + cat	110	61.1

(*n* = 180) ^a^ Multiple responses were allowed; ^b^ Mean ± SD.

**Table 2:** Work equipment specifications in the pet grooming environment.

Equipment Variables	Conditions	Frequency (*N*)	Percentage (%)
Bathing tub depth	<25 cm	3	1.7
	26–35 cm	64	35.6
	36–45 cm	70	38.9
	≥46 cm	43	23.9
Bathing tub height	Below waist	17	9.4
	At waist level	63	35.0
	Between waist and chest	96	53.3
	Above chest	4	2.2
Grooming table height	Fixed (≤74 cm)	23	12.8
	Fixed (75–79 cm)	88	48.9
	Fixed (80–85 cm)	18	10.0
	Adjustable	51	28.3
Hair dryer type	Mini handheld	7	3.9
	Mini with stand	8	4.4
	Wall-mounted large	50	27.8
	Upright stand large	115	63.9

(*n* = 180).

**Table 3:** Analysis of workload data for pet grooming professionals.

Variable	Workload Range	Frequency (*N*)	Percentage (%)
Max daily bathing vol.	10–20	102	56.7
	21–30	51	28.3
	31–40	14	7.8
	≥41	13	7.2
Avg daily bathing vol.	≤8	79	43.9
	9–15	96	53.3
	16–20	2	1.1
	≥21	3	1.7
Daily small dogs	1–5	44	24.4
	6–8	70	38.9
	9–10	40	22.2
	≥10	26	14.4
Weekly medium dogs	0	7	3.9
	1–5	99	55.0
	6–10	52	28.9
	11–15	16	8.9
	≥16	6	3.3
Weekly large dogs	0	29	16.1
	1–3	109	60.6
	4–6	39	21.7
	≥7	3	1.7
Weekly aggressive animals	0	31	17.2
	1–5	114	63.3
	6–10	27	15.0
	≥11	8	4.5
Weekly manual trimming	0	1	0.6
	1–5	124	68.9
	6–10	29	16.1
	11–15	17	9.4
	≥16	9	5.0
Weekly electric clipping	≤5	40	22.2
	6–10	61	33.9
	11–15	49	27.2
	16–20	19	10.6
	≥21	11	6.1

(*n* = 180).

**Table 4:** Prevalence and ranking of musculoskeletal disorders by body region.

Rank	Pain Location	Frequency (*N*)	Percentage (%)
1	Right shoulder	129	71.7
2	Neck	127	70.6
3 (tie)	Upper back	118	62.6
3 (tie)	Lower back	118	65.6
5 (tie)	Left shoulder	103	57.2
5 (tie)	Right hand/wrist	103	57.2
7	Right elbow/forearm	94	52.2
8	Left hand/wrist	79	43.9
9	Left elbow/forearm	76	38.9
10 (tie)	Left knee	42	23.3
10 (tie)	Right knee	42	23.3
12	Left hip/thigh	35	19.4
13	Right hip/thigh	31	17.2
14	Right ankle/foot	29	16.1
15	Left ankle/foot	28	15.6

(*n* = 180).

**Table 5:** Association between work equipment and high-prevalence musculoskeletal disorders.

Equipment Variable	N	Body Region	Frequency (*N*)	Percentage (%)
Tub depth (*n* = 180)				
<25 cm	3	Left shoulder	2	66.7
		Right shoulder	2	66.7
		Neck	1	33.3
26–35 cm	63	Right shoulder	47	74.6
		Upper back	45	71.4
		Neck	44	69.8
		Lower back	37	58.7
36–45 cm	71	Neck	50	70.4
		Right shoulder	49	69.0
		Lower back	46	64.8
≥46 cm	43	Lower back	34	79.1
		Neck	32	74.4
		Right wrist	32	74.4
		Right shoulder	31	72.1
Tub height (*n* = 179)				
Below waist	17	Neck	12	70.6
		Lower back	10	58.8
		Upper back	9	52.9
At waist	62	Right shoulder	42	67.7
		Neck	41	66.1
		Upper back	38	61.3
Waist to chest	96	Right shoulder	75	78.1
		Neck	71	74.0
		Upper back	69	71.9
Above chest	4	Lower back	4	100.0
		Right wrist	4	100.0
		Left knee	3	75.0
Table height (*n* = 176)				
Fixed (≤74 cm)	21	Neck	16	76.2
		Left shoulder	15	71.4
		Upper back	15	71.4
		Lower back	15	71.4
Fixed (75–79 cm)	87	Right shoulder	65	74.7
		Neck	62	71.3
		Upper back	61	70.1
		Lower back	52	59.8
Fixed (80–85 cm)	17	Neck	13	76.5
		Right shoulder	12	70.6
		Lower back	11	64.7
		Right wrist	11	64.7
Adjustable	51	Lower back	38	74.5
		Right shoulder	34	66.7
		Neck	33	64.7
		Upper back	30	58.8

**Table 6:** Work posture and high-prevalence musculoskeletal disorders.

Task & Posture	N	Body Region	Frequency (*N*)	Percentage (%)
Blow-drying (seated)				
Upright (neutral)	66	Right shoulder	46	69.7
		Neck	43	65.2
		Upper back	40	60.6
		Lower back	37	56.1
Neck flexion	64	Right shoulder	46	71.9
		Neck	45	70.3
		Lower back	44	68.8
		Left shoulder	40	62.5
Back flexion	49	Neck	39	79.6
		Right shoulder	37	75.5
		Lower back	37	75.5
		Upper back	34	69.4
Manual trimming				
Seated	71	Neck	56	78.9
		Right shoulder	54	76.1
		Lower back	49	69.0
		Upper back	48	67.6
		Right wrist	44	62.0
Lunge	58	Neck	39	67.2
		Right shoulder	39	67.2
		Lower back	39	67.2
		Upper back	34	58.6
Standing (knees straight)	47	Upper back	36	76.6
		Right shoulder	36	76.6
		Lower back	35	74.5
		Neck	33	70.2
		Left shoulder	33	70.2
Horse stance	37	Right shoulder	31	83.8
		Upper back	26	70.3
		Right wrist	26	70.3
		Neck	25	67.6
Electric clipping				
Seated	136	Right shoulder	102	75.0
		Neck	100	73.5
		Upper back	94	69.1
		Lower back	90	66.2
Lunge	19	Neck	16	84.2
		Lower back	13	68.4
		Right shoulder	13	68.4
		Upper back	12	63.2
Standing (knees straight)	30	Lower back	22	73.3
		Left shoulder	19	63.3
		Right shoulder	19	63.3
		Upper back	18	60.0
Horse stance	13	Right shoulder	11	84.6
		Lower back	10	76.9
		Upper back	9	69.2
		Right wrist	9	69.2

**Table 7:** Relationship between workload and musculoskeletal disorders severity.

Workload	Items	*N*	MSD Score (Mean ± SD)	*p*-Value	Post Hoc
Max daily bathing	a. 10–20	102	8.6 ± 5.9	0.001 **	d > a (*p* = 0.048 *)
	b. 21–30	51	7.9 ± 5.0		d > b (*p* = 0.030 *)
	c. 31–40	14	14.5 ± 13.5		c > a (*p* = 0.027 *)
	d. ≥41	13	14.2 ± 8.9		c > b (*p* = 0.017 *)
Avg daily bathing	a. ≤8	79	7.9 ± 5.4	<0.001 ***	d > a (*p* <0.001 ***)
	b. 9–15	96	9.8 ± 7.4		d > b (*p* <0.001 ***)
	c. 16–20	2	8.0 ± 2.8		d > c (*p* = 0.023 *)
	d. ≥ 21	3	27.0 ± 12.5		
Daily small dogs	a. 1–5	44	6.7 ± 4.9	0.001 **	d > a (*p* = 0.005 **)
	b. 6–8	70	10.2 ± 6.9		d > c (*p* = 0.005 **)
	c. 9–10	40	8.0 ± 6.9		b > a (*p* = 0.07)
	d. ≥ 11	26	12.9 ± 9.0		
Weekly large dogs	a. 0	29	7.8 ± 5.0	0.019 *	
	b. 1–3	109	9.2 ± 7.2		
	c. 4–6	39	9.8 ± 7.1		
	d. 7–9	2	24.5 ± 10.6		
	e. ≥10	1	3.0		
Weekly aggressive	a. 0	31	6.7 ± 4.9	0.041 *	
	b. 1–5	114	9.1 ± 7.1		
	c. 6–10	27	12.0 ± 8.2		
	d. 11–15	5	11.8 ± 5.1		
	e. ≥16	3	13.0 ± 7.5		

(*n* = 180) * *p* < 0.05, ** *p* < 0.01, *** *p* < 0.001.

**Table 8:** Burnout assessment for pet grooming professionals.

Fatigue Type	Classification	Score Range	Frequency (*N*)	Percentage (%)
Personal-related Burnout	Mild	<50	102	56.7
	Moderate	50–70	52	28.9
	Severe	>70	26	14.4
Work-related Burnout	Mild	<45	113	62.8
	Moderate	45–60	45	25.0
	Severe	>60	22	12.2

(*n* = 180).

**Table 9:** Pearson correlation analysis of personal-related and work-related burnout.

	Age	Daily Working Hours	Years of Service	Daily Standing Hours	Daily Sitting Hours	MSD Score
Personal-related Burnout Score	−0.010	0.157 *	0.004	0.038	0.228 **	0.491 ***
Work-related Burnout Score	−0.073	0.142	0.035	0.055	0.194 *	0.428 ***

* *p* < 0.05, ** *p* < 0.01, *** *p* < 0.001.

**Table 10:** Multiple linear regression analysis for the predicting variables of burnout assessment.

Predicting Variables	Not Standardization B	Standardization β	t	Significance (*p* Value)
Personal-related Burnout Score				
(Constant/Intercept)	33.334		2.548	0.012 *
Age	−0.231	−0.103	−0.895	0.372
BMI	0.114	0.024	0.337	0.737
Years of service	0.220	0.084	0.725	0.469
Daily working hours	0.136	0.011	0.148	0.882
Daily standing hours	−0.777	−0.086	−1.155	0.250
Daily sitting hours	1.505	0.169	2.307	0.022 *
MSD Score	1.270	0.469	6.371	<0.001 ***
Work-related Burnout Score				
(Constant/Intercept)	35.134		2.968	0.004 **
Age	−0.247	−0.127	−1.059	0.292
BMI	−0.103	−0.026	−0.338	0.736
Years of service	0.114	0.050	0.416	0.678
Daily working hours	0.288	0.027	0.348	0.729
Daily standing hours	−0.512	−0.066	−0.841	0.402
Daily sitting hours	1.188	0.153	2.011	0.046 *
MSD Score	0.939	0.398	5.200	<0.001 ***

* *p* < 0.05, ** *p* < 0.01, *** *p* < 0.001. Dependent variable: Personal-related Burnout Score/Work-related Burnout Score. Independent variables: Age, BMI, Years of service, Daily working hours, Daily standing hours, Daily sitting hours, and MSD Score.

## Data Availability

The data that support the findings of this study are available from the corresponding author upon reasonable request.
